# Motor unit number index detects the effectiveness of surgical treatment in improving distal motor neuron loss in patients with incomplete cervical spinal cord injury

**DOI:** 10.1186/s12891-020-03567-w

**Published:** 2020-08-15

**Authors:** Jun Li, Yancheng Zhu, Yang Li, Shisheng He, Deguo Wang

**Affiliations:** 1 Department of Orthopedics, College of Clinical Medicine, Shanghai Ten Hospitals of Nanjing Medical University, 301 Yanchang Middle Road, Jing’an District, Shanghai, 200072 China; 2 Department of Orthopedics, Shanghai Songjiang District Central Hospital, Shanghai, 201600 China; 3 Department of Orthopedics, The Second Affiliated Hospital of Nanjing Medical University, Nanjing, 210011 Jiangsu Province China

**Keywords:** Spinal cord injury, Motor unit number index, Motor unit loss, Optimal timing for surgery, Trans-synaptic degeneration

## Abstract

**Background:**

Recovery of motor dysfunction is important for patients with incomplete cervical spinal cord injury (SCI). To enhance the recovery of muscle strength, both research and treatments mainly focus on injury of upper motor neurons at the direct injury site. However, accumulating evidences have suggested that SCI has a downstream effect on the peripheral nervous system, which may contribute to the poor improvement of the muscle strength after operation. The aim of this study is to investigate the impact of early vs. delayed surgical intervention on the lower motor neurons (LMNs) distal to the injury site in patients with incomplete cervical SCI.

**Methods:**

Motor unit number index (MUNIX) was performed on the tibialis anterior (TA), extensor digitorum brevis (EDB) and abductor hallucis (AH) in 47 patients with incomplete cervical SCI (early vs. delayed surgical-treatment: 17 vs. 30) and 34 healthy subjects approximately 12 months after operation. All patients were further assessed by American spinal injury association (ASIA) motor scales and Medical Research Council (MRC) scales.

**Results:**

There are no difference of both ASIA motor scores and MRC scales between the patients who accepted early and delayed surgical treatment (*P* > 0.05). In contrast, the patients undergoing early surgical treatment showed lower MUSIX values in both bilateral EDB and bilateral TA, along with greater MUNIX values in both right-side EDB and right-side TA, compared to the patients who accepted delayed surgical treatment (*P* < 0.05).

**Conclusions:**

Cervical SCI has a negative effect on the LMNs distal to the injury site. Early surgical intervention in Cervical SCI patients may improve the dysfunction of LMNs distal to the injury site, reducing secondary motor neuron loss, and eventually improving clinical prognosis.

## Background

Recovery of motor dysfunction is important for patients with incomplete cervical spinal cord injury (SCI) since it is essential for improving health-related quality of life [1]. To enhance the recovery of muscle strength, both research and treatments mainly focus on both primary and secondary injury of upper motor neurons (UMN) at the direct injury site [2, 3]. In many different neuroprotective treatments, surgical decompression is demonstrated as one of the most important methods, and accumulating evidence has suggested that early operation can relieve both mechanical compression and microcirculation disturbance of cervical cord, therefore improving the clinical prognosis [3, 4].

The pathophysiology of cervical SCI is complex, and recently published studies revealed obvious electrophysiological abnormalities in distal paralyzed muscles in patients with SCI [1, 5–7], suggesting the degeneration of both spinal motoneurons and peripheral motor axons in regions caudal to the level of direct injury, which may contribute to the poor improvement of the muscle strength. Unfortunately, few studies have involved the effects of early surgical treatment on the loss/dysfunction of distal motor neurons, although this issue is important for clinician to establish suitable treatment of cervical SCI.

Motor unit number index (MUNIX) is a recently developed quantitative method that provides an estimated index of the number and size of the functional motor units in the tested muscle [8]. According to the previous studies, MUNIX was demonstrated to be very sensitive in detecting motor unit loss in many different neuromuscular diseases [8–11], and both Li et al. and Marciniak et al. demonstrated MUNIX detection can be used to assess the integrity of lower motor neuron in patients with SCI [12, 13].

The aim of the present study is to quantify the functional motor units in the lower limb muscles in patients with incomplete cervical SCI who accepted early (≤ 72 h) versus delayed (> 72 h) surgical treatment.

## Methods

### Subjects

A total of 47 patients with incomplete cervical SCI and 34 healthy subjects were analyzed retrospectively in this study. In the present study, seventeen patients with cervical SCI underwent early surgical treatment (≤ 72 h), and the other 30 patients underwent delayed surgical treatment (> 72 h) (Table 1). All subjects in cervical SCI patient group were recruited in SongJiang Hospital from December 2015 to June 2017. The study protocol was approved by Human Ethics Committees (Shanghai Songjiang District Central Hospital, KYLL2015–263). All subjects gave informed consent.

**Table 1:** Characteristics of patients with cervical SCI in both surgical treatment groups

	Early surgical treatment group	Delayed surgical treatment group
**Number of subjects**	17	30
**Age range (years)**	45.0 ± 12.2	47.4 ± 13.1
**Height range (cm)**	164.7 ± 8.9	165.9 ± 9.2
**Gender (Male vs. Female)**	12 vs. 5	24 vs. 6
**Time from injury to surgery (days)**	1.9 ± 0.7	17.2 ± 7.9
**Severity of SCI**		
ASIA B	2/17 (11.8%)	2/30 (6.7%)
ASIA C	10/17 (58.8%)	13/30 (43.3%)
ASIA D	5/17 (29.4%)	15/30 (50%)
**Imaging abnormalities (n/total patient (%))**		
Cervical fracture	5/17 (29.4%)	8/30 (26.7%)
Intramedullary high-signal lesion	7/17 (41.2%)	18/30 (60.0%)
**Mechanism of injury (n/total patient (%))**		
Falls	6/17 (35.3%)	9/30 (30.0%)
Vehicle accidents	11/17 (64.7%)	21/30 (70.0%)
**Surgical approach (n/total patient (%))**		
Anterior	5/17 (29.4%)	7/30 (23.3%)
Posterior	13/17 (76.5%)	20/30 (66.7%)
Combined	1/17 (5.9%)	3/30 (10.0%)

*SCI* Spinal cord injury, *ASIA* ASIA (American Spinal Injury Association) impairment scale

The subjects in normal control group were chosen based on the inclusion and exclusion criteria published previously [5]. The inclusion criteria for cervical SCI patients includes (1) a clear history of trauma; (2) different degrees of sensory and motor impairments in both upper limbs and/or the lower limbs with a variable effect on bladder function; (3) magnetic resonance imaging (MRI) and/or Computed tomography (CT) demonstrating SCI at the cervical segment without the injuries in the level of thoracic and lumbar spine injuries. The exclusion criteria for cervical SCI patients includes previous spinal surgery, polyneuropathies, radiculopathies, plexopathies, focal neuropathies, muscle disorders, diabetes, diseases of the central nervous system, syringomyelia, spinal cord tumour/inflammation/infection, spinal deformities and severe degenerative diseases of thoracic and lumbar segments.

### Testing methods

#### Motor unit number index

The MUNIX detection was applied in both 47 patients with incomplete cervical SCI approximately 1 year after operation and 34 healthy subjects. The MUNIX method described by Nandedkar et al. was used in this study [8]. The maximal compound muscle action potential (CMAP) was recorded bilaterally from the tibialis anterior (TA), extensor digitorum brevis (EDB) and abductor hallucis (AH) in a belly-tendon montage (filters: 3 Hz-10 kHz) to supramaximal stimulation. Subsequently, surface interference pattern (SIP) for ten different force levels of isometric contraction was recorded in a 300-ms window (filters: 10 Hz–1000 Hz). According to these measurements, both MUNIX values and motor unit size index (MUSIX) values for these three muscles were measured.

For the evaluation of the reproducibility, left-side MUNIX measurements of 15 healthy subjects and 21 patients with cervical SCI were tested twice by the same examiner. The intervals between these 2 tests were longer than 60 min, and the electrodes were completely removed after the initial test.

All electrophysiological examinations were carried out by Keypoint EMG machine (Medtronic Dantec, Skovlunde, Denmark) with a skin temperature > 32 °C. MUNIX values cannot be measured when the following conditions occur: SIP area < 20 mV.ms, ideal case motor unit count (ICMUC) > 100, SIP area/CMAP area < 1, or CMAP amplitude < 0.5 mV.

### Clinical function examination

All 47 patients with cervical SCI accepted American spinal injury association (ASIA) classification to identify the severity of SCI at the time of admission. All of these patients further underwent muscle strength examination in all tested muscles graded by the Medical Research Council (MRC) scales and American spinal injury association (ASIA) motor scores approximately 1 year after operation.

### Statistical methods

The measurements were analyzed by SPSS 18.0 (IBM, Armonk, NY), and Medsci simple size tools (Shanghai, China) were used to calculate sample size. The measurements among healthy subjects and different cervical SCI patient groups were tested using one-way ANOVA (least significant difference correction). The preoperative and postoperative ASIA motor scores were compared by the paired t-test.

Pearson or Spearman correlation coefficient analysis (CCA) was used to evaluate the relationship between MUNIX values and MRC scales in each patient group. The test-retest reproducibility of MUNIX in healthy subjects and patients with cervical SCI was analyzed using interclass correlation coefficient (ICC) methods.

A *P*-value less than 0.05 was considered significant.

## Results

**Fig. 1:**
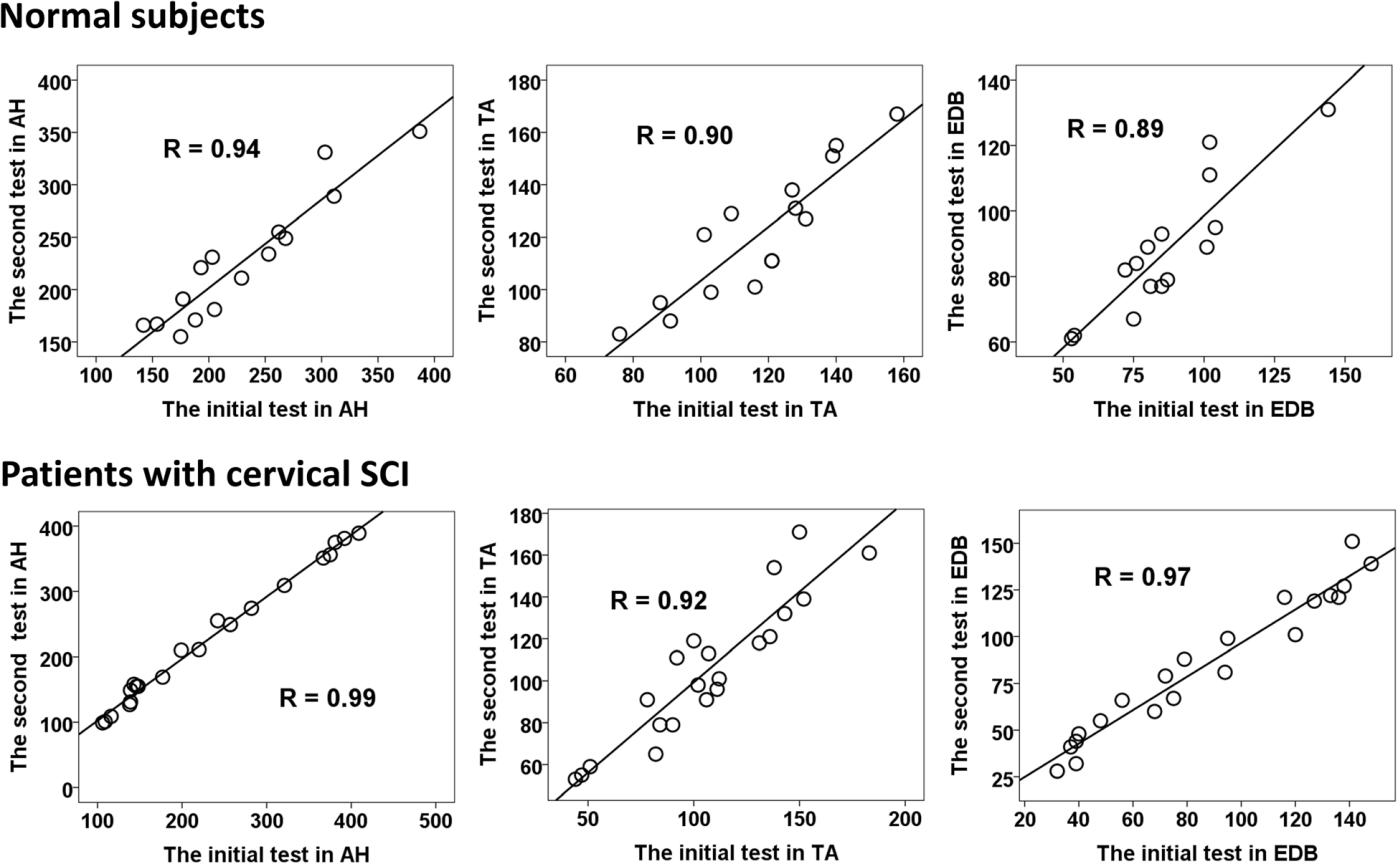
Correlations between MUNIX values of the initial and the second tests in both patients with cervical SCI (left side) and healthy subjects (left side). The graphs show that there is a strong positive correlation between the values of first and the second tests in all tested muscles in both cervical SCI patient and control groups. SCI: Spinal cord injury; MUNIX: motor unit number index; AH: abductor hallucis; EDB: extensor digitorum brevis; TA: tibialis anterior

**Fig. 2:**
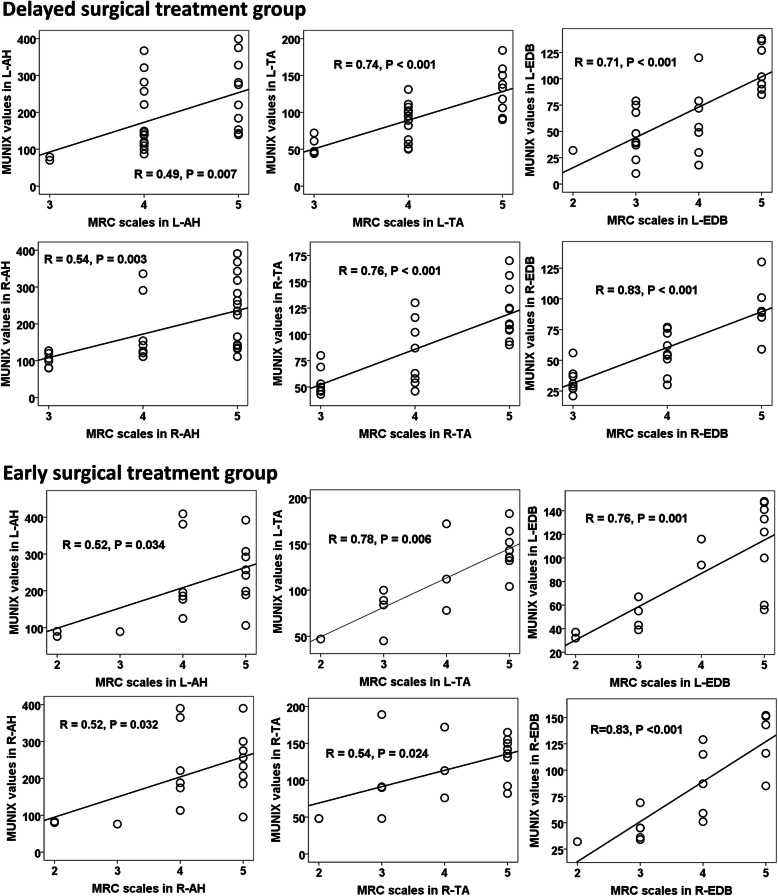
Correlations between MRC scales and MUNIX values in both cervical SCI patient groups. There was a significant relationship between the MRC scales and MUNIX values in all tested muscles in both cervical SCI patient groups. SCI: Spinal cord injury; MRC: medical research council; MUNIX: motor unit number index; AH: abductor hallucis; EDB: extensor digitorum brevis; TA: tibialis anterior; L: left side; R: right side

A significantly positive correlation of all MUNIX values between the initial and second tests was demonstrated in all tested muscles in both normal control and cervical SCI patient groups (*P* < 0.05, Fig. 1), and good reproducibility of all MUNIX measurements was further confirmed by ICC in both subject groups (Table 2). Furthermore, there is an obvious positive correlation between the MUNIX values and MRC scales in all tested muscles in both patient groups (*P* < 0.05, Fig. 2).

**Table 2:** Test-retest reproducibility of MUNIX in patients with SCI and healthy subjects

	Patients with cervical SCI		Healthy subjects	
Number of cases	21		15	
Age range (years)	49.2 ± 11.8		45.0 ± 11.5	
Height range (cm)	163.6 ± 9.2		166.2 ± 6.3	
	ICC	CCA	ICC	CCA
**Abductor hallucis**				
CMAP	0.99	0.99	0.99	0.99
MUNIX	0.99	0.99	0.97	0.94
MUSIX	0.96	0.93	0.96	0.95
**Extensor digitorum brevis**				
CMAP	0.99	0.97	0.98	0.97
MUNIX	0.99	0.97	0.94	0.89
MUSIX	0.95	0.90	0.91	0.84
**Tibialis anterior**				
CMAP	0.99	0.98	0.97	0.94
MUNIX	0.96	0.92	0.94	0.90
MUSIX	0.90	0.84	0.87	0.78

*MUNIX* Motor unit number index, *SCI* Spinal cord injury, *CMAP* Compound muscle action potential, *MUSIX* Motor unit size index, *ICC* Intraclass correlation coefficient, *CCA* Correlation coefficient analysis

In the delayed surgical treatment group, the MUNIX values were not recorded from all tested muscles in two patients. Furthermore, absent MUNIX values were also observed in bilateral TA in one patient and in unilateral EDB in two patients. In addition, the other two patients in this group presented with absent MUNIX values in bilateral EDB, along with absent MUNIX values in left-side TA in one of these 2 patients. Therefore, seven (7/30, 23.3%) patients who accepted delayed surgical treatment presented with absent postoperative MUNIX values in at least one of the tested muscles in this study. In contrast, only one (1/17, 5.9%) patient in the early surgical treatment group presented with absent MUNIX values in bilateral EDB.

Compared with the normal controls, the patients who accepted early surgical treatment presented with increased MUSIX values in both AH and EDB on right side (*P* < 0.05, Table 3). Furthermore, the patients undergoing delayed surgical treatment showed increased MUSIX values in bilateral AH, as well as both reduced MUNIX values and increased MUSIX values in both TA and EDB on bilateral side (*P* < 0.05, Table 3). In addition, delayed surgical treatment group patients also presented with reduced CMAP amplitudes in left-side EDB (*P* < 0.05, Table 3).

**Table 3:** Measurements of MUNIX detection and clinical function measures in both SCI patient and normal control groups

	Early surgical treatment group		Delayed surgical treatment group		Healthy subjects	
**Number of subjects**	17		30		34	
**Age range (years)**	45.0 ± 12.2		47.4 ± 13.1		44.0 ± 11.1	
**Height range (cm)**	164.7 ± 8.9		165.9 ± 9.2		166.9 ± 5.7	
	Left side	Right side	Left side	Right side	Left side	Right side
**MUNIX detection**						
CMAP-AH	14.0 ± 4.7	14.1 ± 4.8	13.4 ± 4.7	13.3 ± 4.5	14.6 ± 4.9	14.2 ± 5.0
MUNIX-AH	218.4 ± 108.3	213.6 ± 106.0	195.9 ± 97.5	192.8 ± 96.0	240.8 ± 96.8	240.4 ± 103.5
MUSIX-AH	71.5 ± 20.3	74.8 ± 24.2*	76.8 ± 14.1*	77.1 ± 14.9*	63.0 ± 12.5	62.7 ± 13.7
CMAP-TA	6.1 ± 1.3	6.1 ± 1.1	5.9 ± 1.8	5.9 ± 1.5	6.5 ± 1.0	6.5 ± 1.0
MUNIX-TA	117.3 ± 41.9	113.4 ± 45.6#	95.3 ± 38.2*	89.6 ± 37.2*#	122.9 ± 31.7	119.6 ± 30.8
MUSIX-TA	58.1 ± 20.3#	61.1 ± 22.2#	70.6 ± 15.6*#	75.7 ± 20.5*#	55.2 ± 10.4	56.4 ± 11.3
CMAP-EDB	5.6 ± 2.2	5.6 ± 2.3	5.0 ± 2.0*	5.0 ± 2.0	6.0 ± 1.5	5.9 ± 1.9
MUNIX-EDB	86.9 ± 42.8	84.3 ± 44.0#	69.6 ± 37.4*	60.4 ± 28.5*#	94.8 ± 30.1	94.7 ± 34.4
MUSIX-EDB	73.8 ± 19.2#	76.7 ± 18.3*#	87.7 ± 30.2*#	92.9 ± 20.0*#	65.2 ± 13.6	63.7 ± 12.2
**Clinical function measures**						
MRC-AH	4.2 ± 1.0	4.2 ± 1.0	4.1 ± 0.8	4.2 ± 1.0	/	/
MRC-TA	3.9 ± 1.2	4.0 ± 1.1	3.9 ± 1.0	3.9 ± 1.0		
MRC-EDB	3.8 ± 1.3	3.7 ± 1.2	3.7 ± 1.0	3.7 ± 1.0	/	/
ASIA motor score	81.4 ± 15.2		78.6 ± 18.3		/	

*SCI* Spinal cord injury, *MUNIX* Motor unit number index, *CMAP* Compound muscle action potential, *MUSIX* Motor unit size index, *TA* tibialis anterior, *AH* abductor hallucis, *EDB* Extensor digitorum brevis, *MRC* Medical research council score, *ASIA* American spinal injury association

* Statistical difference between the patient and control groups, *P* < 0.05

# Statistical difference between the early and delayed surgical treatment groups, *P* < 0.05

There is no difference of ASIA motor scores between the patients who accepted early and delayed surgical treatment, and MRC scales in all tested muscles were similar between the early and delayed surgical treatment groups (*P* > 0.05, Table 3). In contrast, the patients undergoing early surgical treatment showed lower MUSIX values in both bilateral EDB and bilateral TA, along with greater MUNIX values in both right-side EDB and right-side TA, compared to the patients who accepted delayed surgical treatment (*P* < 0.05, Table 3).

## Discussion

The MUNIX method used in this study only requires a few numbers of electrical stimulation, and it usually takes approximately 30 min to measure six muscles in one subject, thus facilitating patient compliance. More importantly, according to the results of both ICC and CCA, we identified the excellent levels of reproducibility of MUNIX measurements in both the control and SCI patient groups. Therefore, the findings of this study are technically sound and reflect reliably functional motor units of the tested muscles.

In the present study, significant loss of motor units in the lumbosacral-innervated muscles in patients with cervical SCI, suggesting that peripheral nerve function is compromised following upper motor neuron lesion. Central disconnection of the second-order motor neurons is considered to be the likely reason for this condition, and many previous studies have provided evidence with regard to this disconnection may cause loss of lower motor neurons (LMNs) distal to the initial injury site, which may cause the motor axonal degeneration and an eventual reduction in motor units [1, 5, 12–14].

Although the measurements of MUNIX detection were obvious difference between the patients who accepted early and delayed surgical intervention approximately 1 year after operation, both CMAP amplitudes in both AH and TA and MRC scores in all tested muscles were similar between these two patient groups. This condition may be ascribed to these evaluation methods are relatively suboptimal to detect subtle difference between different treatments. Similar results were also reported in many previous studies [8, 11, 15–17], and collateral sprouting induced by longstanding neurodegeneration is likely the main reason. The remarkable increase in MUSIX values in the tested muscles in this study further identified the existence of the reinnervation from the surviving motor axons. According to previous studies, the reinnervation process can provide a functional compensatory mechanism to preserve muscle strength, and recently published study further indicated muscle strength usually can be preserved by reinnervation until 50% or more of motor units are lost [1, 18, 19]. Therefore, compared to current clinical biomarkers (e.g., CMAP amplitudes and muscle strength), MUNIX may be a more objective, sensitive and reliable method for monitoring the treatment outcomes of SCI. Although clinical function improvement is very important to the patients with SCI, the difference of MUNIX measurements between two different treatment group may provide additional unique information for guiding the doctors to select treatment modalities.

When reviewing these findings, one of the limitations is that MUNIX detection is easily influenced by both the examiner and the protocol (e.g., recording of maximum CMAP amplitude). Thus, a standard protocol and a fixed examiner were used in the current study [8]. Furthermore, another limitation is that the time cutoffs by which to identify early vs. delayed surgical treatment were different in various studies involving SCI (e.g., 12 h, 24 h, 4 days and 2 weeks) [20, 21]. Although the definition of early surgery as within the first 2 weeks is not the most common time cutoff, the recent systematic review demonstrated it is preferable to operate within 2 weeks compared to operation within 24 h [22]. Furthermore, few patients can accept surgical treatment within 24 h, due to both transportation issues and referral delays. The other clinical limitation of this study is low sample size, which may be ascribed to the fewer patients with incomplete cervical SCI have complete medical records and more patients lost to follow-up. More significant results might be achieved in future studies with an increased number of both cases and sub-groups.

## Conclusion

Cervical SCI has a potential negative effect on the motor neurons distal to the level of injury. Equally important, different follow-up results of the MUNIX detection between the early vs. delayed surgical treatment group supported that early surgical intervention in some patients with SCI may relatively improve the dysfunction of LMNs and reduce the loss of motor neurons, eventually improving clinical prognosis.

## Data Availability

The datasets used and/or analysed during the current study are available from the corresponding author on reasonable request.

## Abbreviations

SCI: Spinal cord injury
UMN: Upper motor neuron
LMN: Lower motor neuron
MUNIX: Motor unit number index
TA: Tibialis anterior
EDB: Extensor digitorum brevis
AH: Abductor hallucis
CMAP: Compound muscle action potential
SIP: Surface interference pattern
MUSIX: Motor unit size index
MRC: Medical Research Council
ASIA: American spinal injury association
CCA: Correlation coefficient analysis
ICC: Interclass correlation coefficient
